# The Effect of Advance Care Planning on Heart Failure: a Systematic Review and Meta-analysis

**DOI:** 10.1007/s11606-019-05482-w

**Published:** 2019-11-12

**Authors:** Markus Schichtel, Bee Wee, Rafael Perera, Igho Onakpoya

**Affiliations:** 1 Department of Public Health and Primary Care, Primary Care Unit, University of Cambridge, Cambridge, UK; 2 Oxford Centre for Education and Research in Palliative Care, Churchill Hospital, Oxford, UK; 3 Nuffield Department of Primary Care Health Sciences, University of Oxford, Oxford, UK

**Keywords:** heart failure, palliative care, advance care planning, effect, outcomes, systematic review, meta-analysis

## Abstract

**Background:**

Advance care planning is widely advocated to improve outcomes in end-of-life care for patients suffering from heart failure. But until now, there has been no systematic evaluation of the impact of advance care planning (ACP) on clinical outcomes. Our aim was to determine the effect of ACP in heart failure through a meta-analysis of randomized controlled trials (RCTs).

**Methods:**

We searched CINAHL, Cochrane Central Register of Controlled Trials, Database of Systematic Reviews, Embase, ERIC, Ovid MEDLINE, Science Citation Index and PsycINFO (inception to July 2018). We selected RCTs including adult patients with heart failure treated in a hospital, hospice or community setting. Three reviewers independently screened studies, extracted data, assessed the risk of bias (Cochrane risk of bias tool) and evaluated the quality of evidence (GRADE tool) and analysed interventions according to the Template for Intervention Description and Replication (TIDieR). We calculated standardized mean differences (SMD) in random effects models for pooled effects using the generic inverse variance method.

**Results:**

Fourteen RCTs including 2924 participants met all of the inclusion criteria. There was a moderate effect in favour of ACP for quality of life (SMD, 0.38; 95% CI [0.09 to 0.68]), patients’ satisfaction with end-of-life care (SMD, 0.39; 95% CI [0.14 to 0.64]) and the quality of end-of-life communication (SMD, 0.29; 95% CI [0.17 to 0.42]) for patients suffering from heart failure. ACP seemed most effective if it was introduced at significant milestones in a patient’s disease trajectory, included family members, involved follow-up appointments and considered ethnic preferences. Several sensitivity analyses confirmed the statistically significant direction of effect. Heterogeneity was mainly due to different study settings, length of follow-up periods and compositions of ACP.

**Conclusions:**

ACP improved quality of life, patient satisfaction with end-of-life care and the quality of end-of-life communication for patients suffering from heart failure and could be most effective when the right timing, follow-up and involvement of important others was considered.

## BACKGROUND

Regardless of therapeutic advances, heart failure remains an unpredictable, progressive and ultimately fatal long-term condition.^1, 2^ Existing therapies delay, but generally tend not to reverse disease progression.^3^ Consequently, the prevalence of symptomatic heart failure has increased, including a prolongation of the refractory phase of the disease.^4^ An ageing population, a rising prevalence and new life-prolonging treatment approaches mean that over 5% of patients suffering from heart failure have developed treatment refractory symptoms.^5^ There is widespread recognition that these patients merit support from specialist supportive and palliative care services.^6–10^

Advance care planning (ACP) is widely advocated to improve quality of life and patient satisfaction with end-of-life care in heart failure and a reduction of stress and depression in surviving relatives.^5, 7, 11, 12^ ACP is defined as a voluntary process of discussion between healthcare professionals, patients, family and important others to identify patient’s preferences if the patient may lack the competence to make such decisions in the future.^13, 14^ ACP can be a chance for patients to describe what they want to happen and what they do not want to happen in the future. For example, ACP supports patients by clarifying their resuscitation preferences in case of a cardiac arrest, whether or not or under what circumstances to disable an implantable cardioverter-defibrillator (ICD), choosing a healthcare proxy, deciding on their preferred place of care or making decisions on artificial hydration.^13^ ACP can take the shape of a structured discussion or result in the documentation of care preferences in medical records that are regularly reviewed and updated. The context of ACP can be a nurse or consultant-led heart failure outpatient clinic, during inpatient care, or in the course of an appointment with a primary care clinician.

While the use of ACP is promoted to provide better clinical outcomes, its effectiveness to achieve that aim has not been systematically assessed.^15^ The majority of systematic reviews on ACP are concerned with the effect of interventions that promote the *implementation* of ACP and assess outcomes like the rate of ACP conversations or the completion of ACP documents.^16–21^ The systematic literature reviews that investigate the impact of ACP on end-of-life care outcomes combine qualitative and quantitative studies, do not conduct a meta-analysis and do not focus on the effect of ACP on heart failure.^22–27^ The aim of this study was to undertake a systematic review and meta-analysis of RCTs to determine whether end-of-life care which included ACP resulted in improved outcomes for patients suffering from heart failure compared to end-of-life care which did not incorporate ACP. Our hypothesis was that ACP improved clinical outcomes for patients suffering from heart failure. Our main research question was “What is the effect of ACP on clinical outcomes for patients suffering from heart failure?”

The objectives were as follows:

- To determine the effect of advance care planning on the quality of life of patients suffering from heart failure, their satisfaction with end-of-life care through random effects meta-analyses
- To conduct sensitivity analyses and investigate causes of clinical heterogeneity
- To explore characteristics of advance care planning interventions that are associated with high and low effect sizes

## METHODS

We conducted this review in accordance with the Preferred Reporting Items for systematic reviews and meta-analysis (PRISMA-P),^28^ the Cochrane Collaboration reporting items for systematic reviews and meta-analysis^29^ and the Grading of Recommendation Assessment, Development, and Evaluation (GRADE) for the quality of evidence in selected trials.^30^ Intervention impact was reported based on CONSORT guidelines.^31^ Additional File (AF) 1 shows the review protocol.

### Study Selection

We included only randomized controlled trials (RCTs) and cluster randomized controlled trials (cRCTs). No restrictions were placed on the healthcare setting, context or healthcare professional involved. Patients were eligible if they suffered from heart failure defined as a complex syndrome in which the ability of the heart to maintain the circulation of blood is impaired as a result of a structural or functional impairment of ventricular filling or ejection.^5^ Symptoms were classified according to severity using the New York Heart Association (NYHA) functional classification as found in medical records or by a clinician’s diagnosis.^32^ Patients were seen in primary or secondary care, living in the community or nursing homes. We did exclude studies involving people under the age of 18 years and suffering from congenital heart disease.^33^

As defined in policy documents and by experts, we included all types of ACP interventions that provided a coordinated and comprehensive approach of care for patients early, during or towards the end of suffering from heart failure.^33–35^ Authors had to state explicitly ACP intentions or this had to be evident in the composition of the intervention of the study. Interventions that only dealt with do-not-attempt cardio-pulmonary-resuscitation were excluded from the study because they on their own did not represent the complexity of ACP.^36^

We followed the recommendation of the European Association for Palliative Care for the selection of outcome measures^37^ including patient-reported outcome measures that had been validated with heart failure patients requiring palliative care.^38^ Therefore, we selected a priori as our primary outcome quality of life. As secondary outcomes, we chose patient satisfaction with end-of-life care and the quality of end-of-life communication between clinician and patient. Outcomes had to be measured through instruments that had psychometric properties.

### Data Sources and Searches

Together with a specialist health science librarian (NR), we used filters to reliably identify RCTs and searched the following data bases from their inception until 13 July 2018: CINAHL, Cochrane Central Register of Controlled Trials, Cochrane Database of Systematic Reviews, Database of Abstracts of Reviews of Effects, Embase, ERIC, Ovid MEDLINE (SP), Other Non-Indexed Citations and Ovid MEDLINE(R), Science Citation Index, Social Science Citation Index & Conference Proceedings and PsycINFO. A MEDLINE search strategy is presented in AF 2. We checked the reference lists of 11 relevant reviews^16–25, 39^ and all potential cross references. We contacted authors of main trials and experts in the field who were known to conduct research for additional papers or to provide missing or unpublished study data.

### Data Extraction and Quality Assessment

Titles and abstracts were independently screened against inclusion criteria by two reviewers (MS, IO). Disagreements were resolved by discussion or referred for arbitration to a third reviewer (RP). Full texts of screened papers were assessed for inclusion criteria and study quality. We pilot tested eligibility criteria and included a flow diagram of study selection and reasons for exclusion to conform to the PRIMSA statement.

We assessed the overall quality of the trials’ methods by using the GRADE system Version 3.6.1.^40^ A GRADE profile was created for each pooled estimate, for each outcome and for single trials comparing ACP with standard care for each end-of-life outcome. Two authors (MS, IO) independently extracted data from papers and documented findings. AF 3 gives an example of a data extraction form.

### Data Synthesis and Analysis

We performed quantitative meta-analysis with RevMan 5.3.5^41^ using random effects models. For individual studies with continuous outcomes measured by a variety of scale instruments, we calculated standardized mean differences (SMDs) with 95% confidence intervals (CI) between the intervention and control group as recommend by Cochrane.^29^ The effect of SMDs was interpreted as follows: < 0.2 = small effect; between 0.2 and 0.5 moderate effect; 0.5 or higher = large effect.^29^ We re-expressed SMDs to odds ratios [log(OR) = SMD × pi / sqrt^3^] to aid clinical interpretation as recommended by the Cochrane Handbook.^29^ We did not adjust sample sizes to account for clustering in the included cRCT^42^ as this study reported adjusted effect estimates, which took the intra-class coefficient found into consideration. We used a generic inverse variance approach and random effects meta-analysis to include the estimate into the meta-analysis. When multiple time points were reported, we used the one closest to 3 months post-intervention based on research practice in palliative care^43^ and the premise that it would require a reasonable length of time for ACP to take effect.

We assessed heterogeneity using the *I*^2^ statistic stating the percentage of variability in effect estimates that is due to heterogeneity rather than to chance.^44^ Thresholds for the interpretation of heterogeneity were based on Cochrane guidance^29^ as follows: 0% = no heterogeneity; > 0 to 40%: might not be important; 30 to 60%: moderate heterogeneity; 50 to 90%: substantial heterogeneity; 75 to 100%: considerable heterogeneity. We investigated clinical heterogeneity by pre-specified subgroup analyses for each outcome stratified for patient population, length of follow-up periods and study setting. We compared interventions that included only ACP and interventions that included ACP as part of a palliative care programme. We analysed the characteristics of ACP interventions that resulted in either high or low effect sizes for each outcome and explored their content, timing and frequency and context of the intervention according to the Template for Intervention Description and Replication (TIDieR).^45^

We assessed publication bias by investigating the funnel plot symmetry and performing Egger’s test^46^ with STATA version 14.^47^ Two reviewers (MS, IO) independently assessed the risk of bias using the Cochrane Collaboration’s tool for risk of bias domains.^48^

## RESULTS

**Figure 1:**
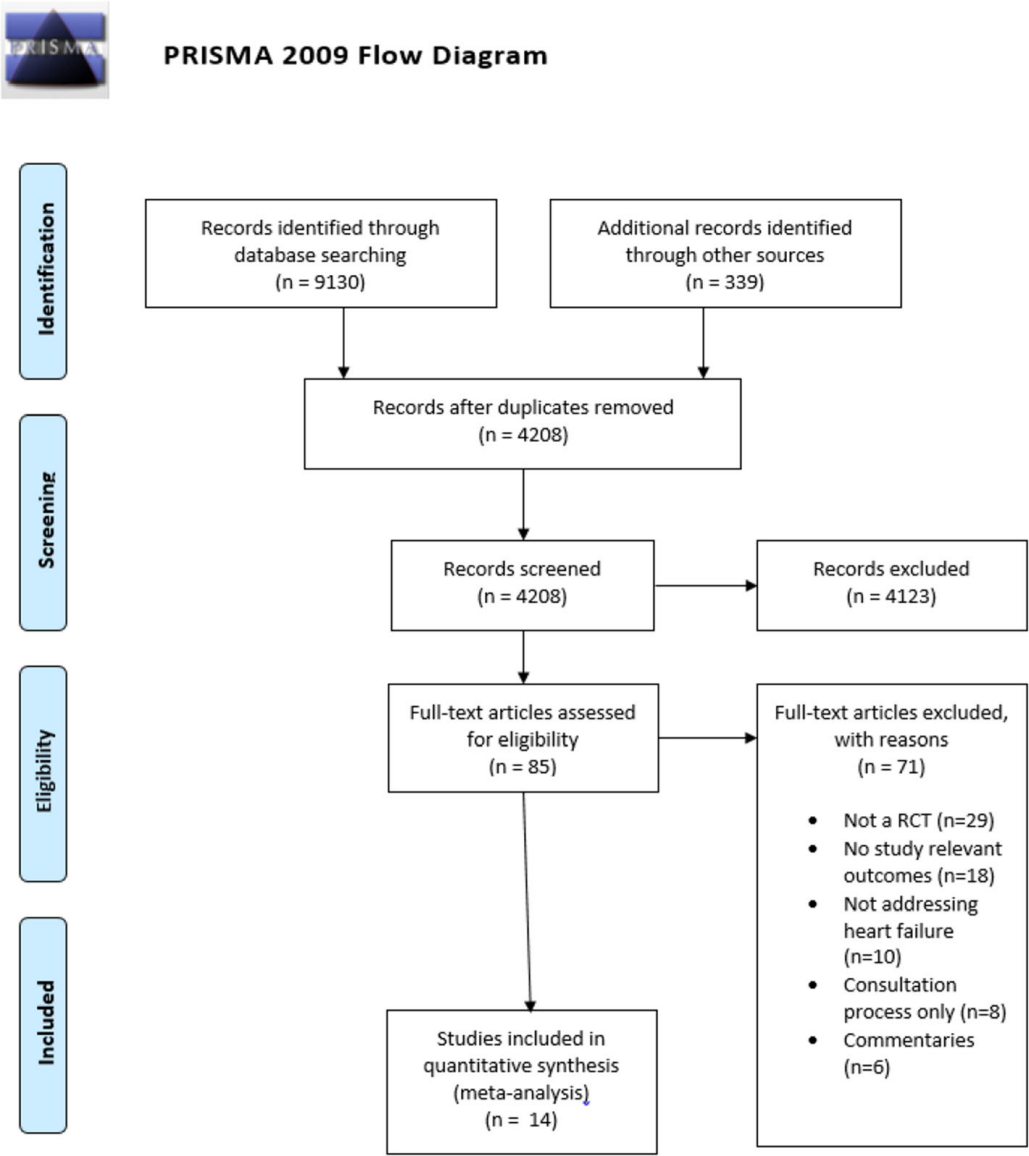
PRISMA flow diagram

Of 9130 articles screened, we reviewed the full text of 85 studies. Fourteen studies (13 RCTs, one cRCTs) met all of the inclusion criteria for the quantitative analysis. Figure 1 shows the PRISMA flow diagram. A detailed description of each trial is provided in Table 1. We included 14 studies in the meta-analysis involving 2924 unique participants. We excluded 71 studies with reasons provided in AF 4 “Characteristics of Excluded Studies.”

**Table 1:** Summary of Included Studies

Study (years)	Country	Setting	Speciality	Description of healthcare professionals	Description of patients	Patients randomized/completed	Female patients (no/%) / mean age (years)	Follow-up (weeks)	Type(s) of outcome(s)
Aiken^49^ (2006)	USA	Community	Palliative Care	Hospice nurse and case manager	HF-NYHA III, IV and COPD	192/191	123/64 68.5	12	QOL
Au^42^ (2012)	USA	Hospital	Medicine	Internal and pulmonary physicians	HF-NYHA III, IV, COPD, CRF	376/306	149/53.26 69.4	2	QEOLC
Brannstrom^50^ (2014)	Sweden	Hospital	Geriatrics	Geriatricians	CHF, NYHA III–IV	72/61	21/29.1 81.9	12	QOL
Briggs^51^ (2004)	USA	Hospital	Medicine	Cardiologists, renal physicians	HF-NYHA II, III, IV and CRF	27/27	11/40.74 68.7	1	QEOLC
Brumley^52^ (2007)	USA	Community	Primary and Palliative Care	Primary care and palliative care clinicians	HF-NYHA III–IV, COPD, cancer	310/297	146/49 65.1	12	PSEOLC
Denvir^53^ (2016)	UK	Hospital	Cardiology	Cardiology staff	Patients with HF-NYHA III, IV and ACS	50/44	20/40 81.05	12	QOL
Detering^54^ (2010)	Australia	Hospital	Medicine Cardiology	Internal and pulmonary physicians, cardiologists	Elderly HF patients >80 years of age	309/305	162/52.5 84.5	12	PSEOLC
Doorenbos^55^ (2016)	USA	Hospital	Cardiology	Cardiology staff	HF-NYHA I, II, III, IV	80/73	19/23.7 58.1	2	QEOLC
Engelhardt^56^ (2006)	USA	Hospital and community	Primary care and medicine	Primary and secondary care physicians	HF-NYHA III, IV, COPD, cancer	275/186	118/82.6 Not reported	12	PSEOLC
Gade^57^ (2008)	USA	Hospital	Medicine	Internal physicians	HF, cancer, COPD, stroke, CRF	517/512	162/59 73.3	24	PSEOLC
Hopp^58^ (2016)	USA	Hospital	Palliative care	Palliative care clinician and nurse practitioner	CHF patients	85/85	41/48.2 68.1	12	QOL
Rogers^59^ (2017)	USA	Hospital	Palliative care cardiology	Palliative care clinicians, cardiologists	Patients with HF-NYHA III, IV	150/ 106	71/47.3 71.9	12	QOL
Sidebottom^60^ (2015)	USA	Hospital	Cardiology	Cardiology staff	HF patients	232/ 167	110/47.4 73.4	12	QOL
Wong^61^ (2016)	Hong Kong	Hospital	Palliative care	Palliative care physicians and nurses	Patients with HF-NYHA III, IV	84/ 84	41/48.8 78.3	12	QOL

*ACS* acute coronary syndrome, *CHF* congestive heart failure, *COPD* chronic obstructive pulmonary disease, *CRF* chronic renal failure, *HF* heart failure, *NYHA* New York Heart Association, *PSEOLC* patient satisfaction with end-of-life care, *QEOLC* quality of end-of-life communication, *QOL* quality of life

### Characteristics of Included Studies

Ten of the 14 studies were conducted in the USA, one in the UK,^53^, one in Australia,^54^ one in Sweden^50^ and one in Hong Kong.^61^ The median sample size was 209 people (range 27 to 517 participants). The median age of participants was 65.85 (age range 58.1–84.5 years). 40.83% (*n* = 1194) of the total study population were female.

A summary of a risk of bias table and a risk of bias ratings for each study and each trial arm is included in AF 5. Allocation concealment (selection bias) was unclear in seven studies as authors did not describe methods of concealment. Blinding of participants was not possible in seven trials because of the nature of the interventions. One study had a high risk of detection bias since outcome assessors were aware of the participants’ allocation to the intervention group.^56^

### Effects of ACP on Outcomes for Heart Failure

AF 6 shows the GRADE quality ratings for each outcome. The mean score for the overall quality of evidence across all studies was low to moderate. This was mainly due to the fact that many participants could not be blinded to the nature of the intervention introducing a high risk of performance bias.

### Meta-analysis on ACP and Quality of Life

**Figure 2:**
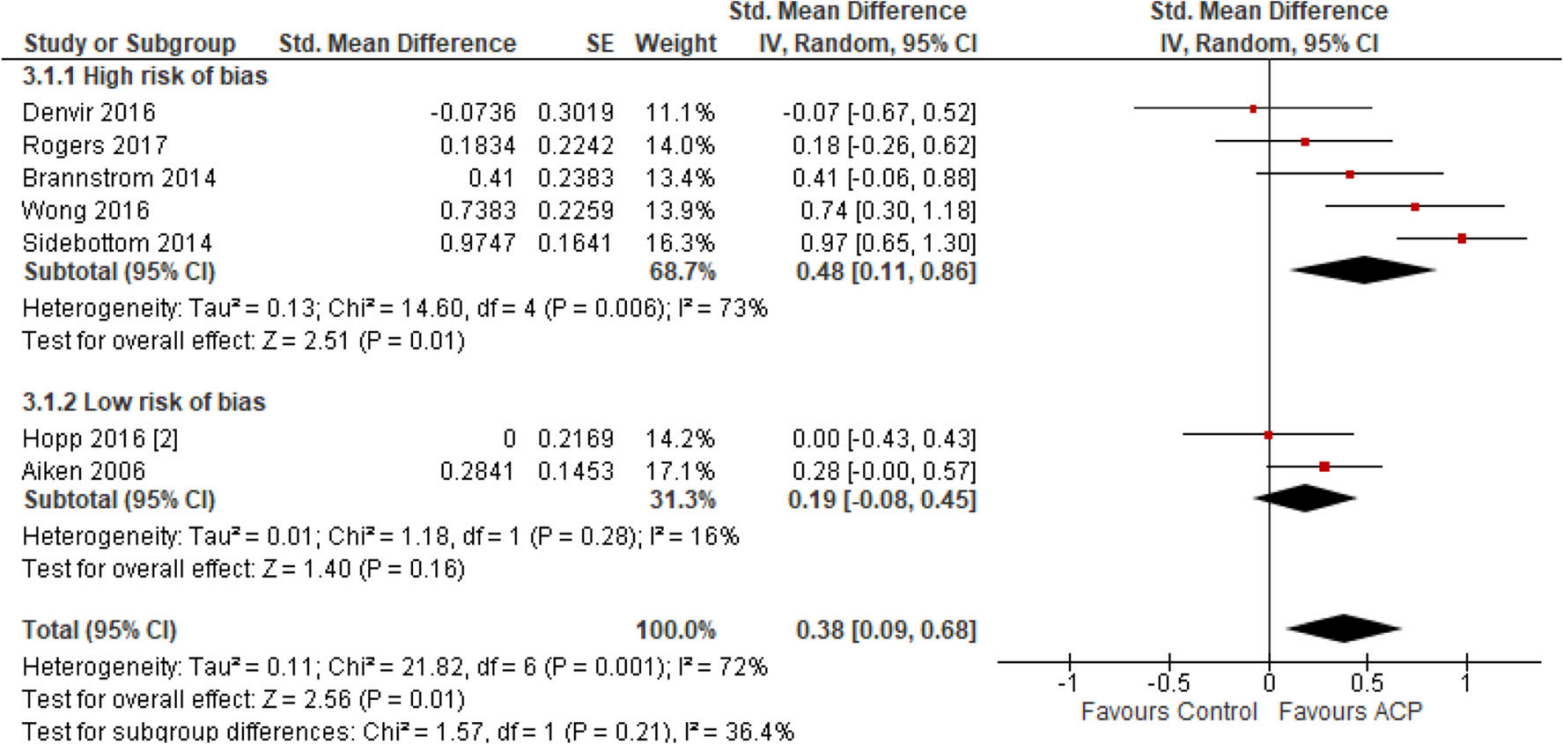
Effect of ACP on quality of life**.**

Quality of life (QOL) was determined in seven studies (724 patients) (Fig. 2). ACP was associated with a statistically significant improvement for QOL (SMD, 0.38; 95% CI [0.09 to 0.66], *p* < 0.009). The median follow-up duration was 12 weeks. Egger’s test did not show any evidence (*p* = 0.406) of a small study effect on publication bias (AF 7).

### Meta-analysis on ACP and Patient Satisfaction

**Figure 3:**
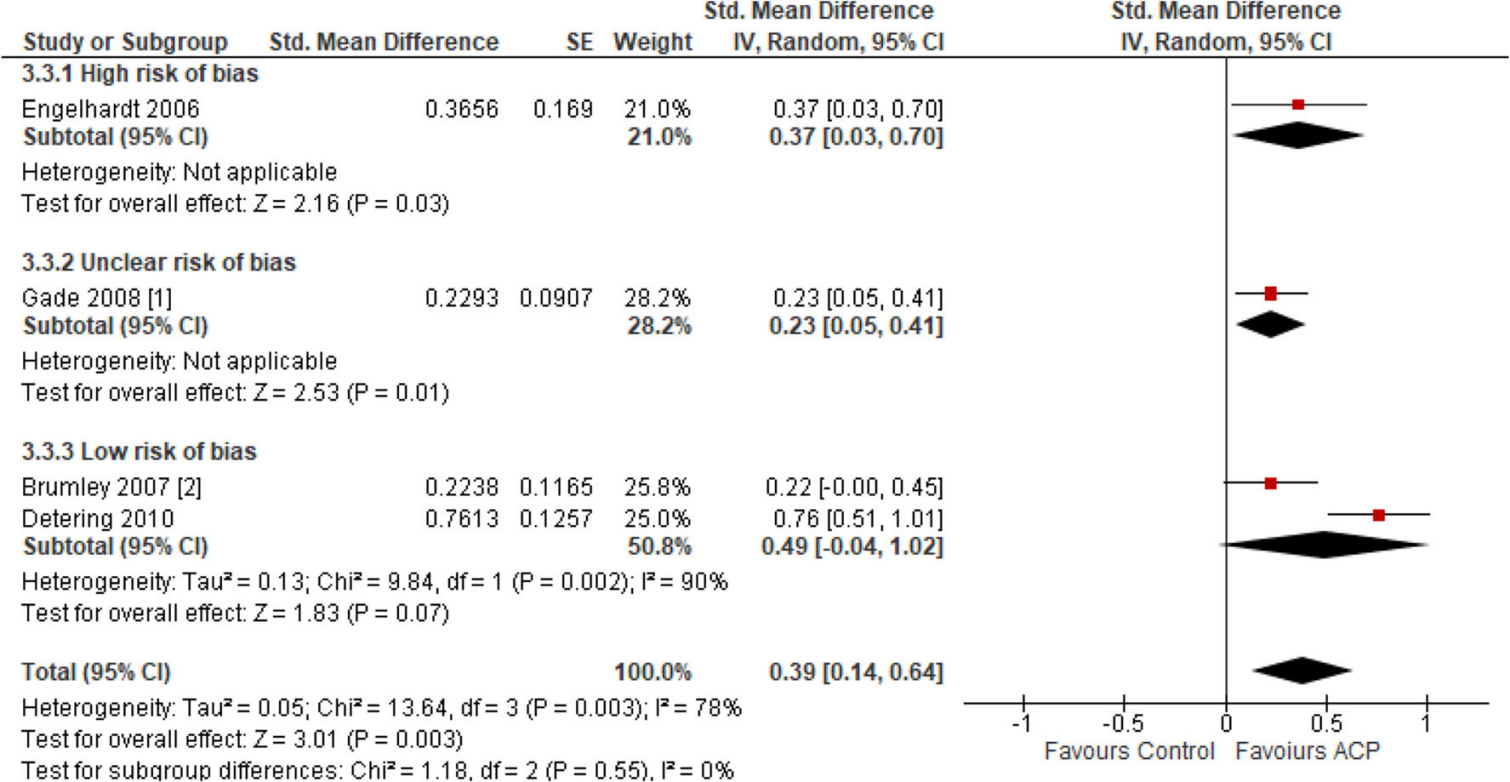
Effect of ACP on patient satisfaction**.**

Patient satisfaction with end-of-life care was assessed in four studies (1290 patients). ACP was associated with (Fig. 3) a statistically significant effect (SMD, 0.39; 95% CI [0.14 to 0.64]; *p* = 0.003). The median follow-up duration was 14.4 weeks. Egger’s test did not show any evidence (*p* = 0.584) of a small study effect on publication bias (AF 7).

### Meta-analysis on ACP and Quality of End-of-Life Communication

**Figure 4:**
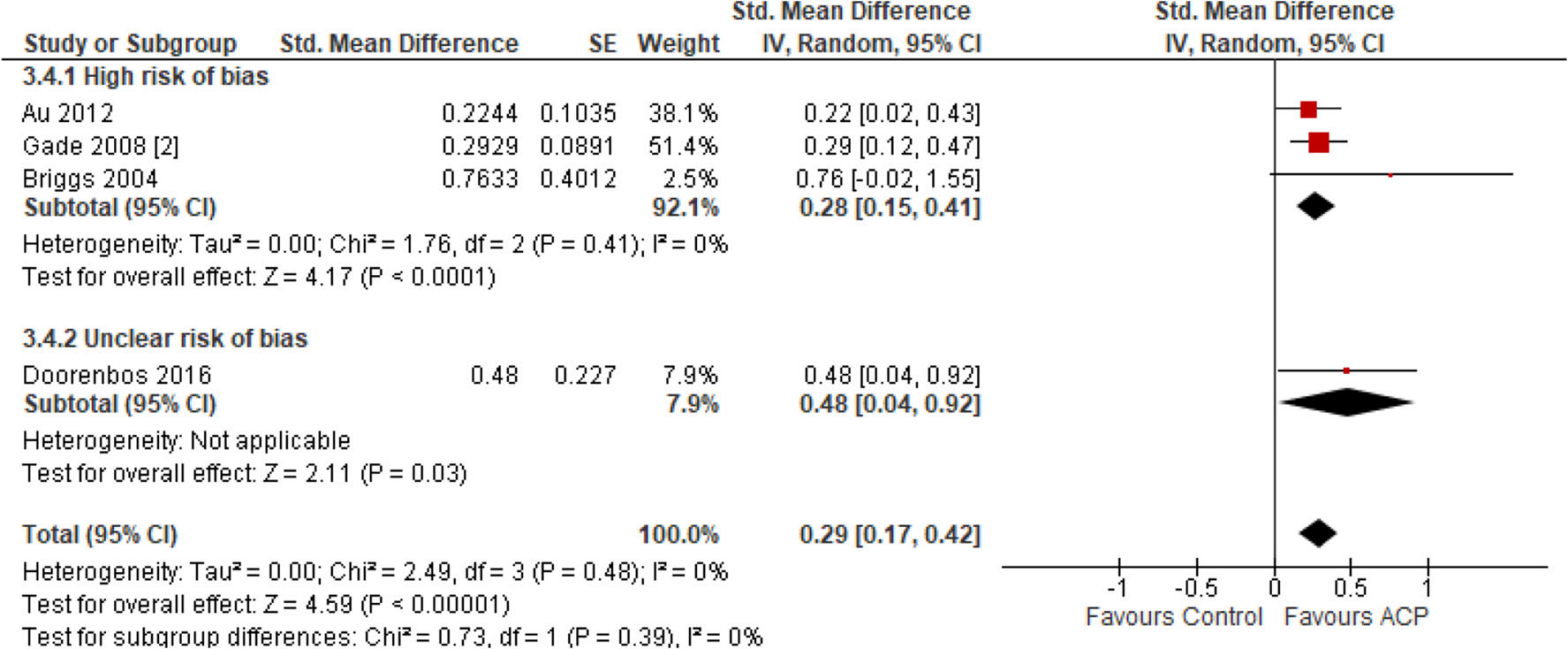
Effect of ACP on quality of communication**.**

Four studies measured the effect of ACP on the quality of end-of-life communication (995 patients). ACP was associated with statistically significant improvement (Fig. 4) for the quality of end-of-life communication (SMD, 0.29; 95% CI [0.17 to 0.42]; *p* < 0.001). The median follow-up duration was 7.25 weeks. Egger’s test of the 4 RCTs did not show any evidence (*p* = 0.095) of a small study effect on publication bias (AF 7).

To aid the clinical interpretation of the results, we re-expressed estimated SMDs for all outcomes as odds ratios according to Cochrane guidelines (AF 8).^29^ ACP improved the quality of life for heart failure patients by approximately 93%, patient satisfaction with end-of-life care by about 96% and the quality of end-of-life communication by around 52%.

### Characteristics of ACP Interventions with High and Low Effect Sizes

Table 2 summarizes the characteristics of ACP interventions with the highest and lowest effect sizes by outcome. Generally, ACP interventions that included multiple components, educated patients, involved family members, offered follow-up and considered ethnic preferences were more effective than single component interventions that did not educate patients, did not involve family members and did not offer follow-up.

**Table 2:** Characteristics of ACP Interventions

Study	Outcome	Effect size SMD [95% CI]	ACP characteristics	ACP timing	Education of patient	Involvement of family	ACP follow-up
Sidebottom (2014)	QOL	0.94 [0.62 to 1.26]	A trained facilitator supports patients to identify their care preferences, completes an ACP health directive and a HF disease-specific care plan	At hospital admission	Yes	Yes	Yes
Wong (2016)	QOL	0.74 [0.30 to 1.18]	A complex ACP and transitional palliative care programme with interdisciplinary communication	Before hospital discharge	Yes	Yes	Yes
Hopp (2016)	QOL	0.00 [− 0.43 to 0.43]	Single component ACP intervention: one meeting to complete an ACP document, no further patient support	During hospital stay	No	No	No
Denvir (2016)	QOL	− 0.07 [− 0.67 to 0.52]	An ACP document is discussed with patients, using a multidisciplinary approach and patient electronic records	Before hospital discharge	Unclear	Unclear	Yes
Detering (2010)	PSEOLC	0.76 [0.52 to 1.01]	A complex respecting patient choices programme including ACP, identification of patient’s care preferences and surrogate decision maker	Before hospital discharge	Yes	Yes	Yes
Engelhardt (2006)	PSEOLC	0.37 [0.03 to 0.70]	A complex ACP coordinated care programme: training patients to ask questions, multidisciplinary approach, increasing patient self-management	Patients were stable	Yes	Yes	Yes
Brumley (2007)	PSEOLC	0.22 [− 0.00 to 0.45]	An interdisciplinary, home-based ACP programme including patients’ self-management	Patients were unwell	Unclear	Yes	Yes
Doorenboos (2016)	QEOLC	0.48 [0.03 to 0.92]	Pre-outpatient telephone call to train patients in ACP communication and identifying end-of-life care wishes; clinician informed of patient’s wishes before visit	Hospital outpatient visit	Yes	Yes	Yes
Briggs (2004)	QEOLC	0.76 [− 0.02 to 1.55]	A single ACP intervention: facilitator has a single 2-h ACP meeting with patient, no interdisciplinary working and no F/u	Before elective admission to hospital	Yes	Yes	No

*ACP* advance care planning, *F/u* follow-up, *HF* heart failure, *PSEOLC* patient satisfaction with end-of-life care, *QEOLC* quality of end-of-life communication, *QOL* quality of life

### Sensitivity Analyses and Heterogeneity

Several sensitivity analyses (AF 9) including studies restricted to ACP only (SMD, 0.35; 95% CI [− 0.10 to 0.81]) versus ACP plus palliative care programme (SMD, 0.32; 95% CI [0.16 to 0.48]), US-based studies (SMD, 0.37; 95% CI [− 0.05 to 0.79]) versus not US-based studies (SMD, 0.39; 95% CI [− 0.04 to 0.83]), and trials at low risk of bias (SMD, 0.34; 95% CI [0.03 to 0.65]) confirmed the consistency of the results of the primary analyses.

To investigate possible causes of heterogeneity, additional sensitivity analyses were performed by stratifying outcomes for patient populations, study settings and length of follow-up periods. No heterogeneity was detected for the hospital setting for quality of life, for the quality of end-of-life communication and for heart failure and other terminally ill patients for the outcome quality of end-of-life communication. Levels of heterogeneity seemed lower for shorter follow-up periods (1 to 4 weeks) than for longer follow-up durations (up to 12 or 24 weeks). Quality of life and patient satisfaction with end-of-life care showed higher levels of heterogeneity than quality of end-of-life communication. A summary of causes of heterogeneity for each outcome and subgroup is presented in Table 3. These differences are explored in more detail under the “Discussion” section.

**Table 3:** Causes of Heterogeneity

Outcome and subgroups	Studies	Participants	Effect size SMD, 95% CI	*I*^2^
Quality of life (QOL)	7	724	0.38 [0.09 to 0.66]	71%
Patient population				
QOL HF patients	6	532	0.39 [0.04 to 0.74]	74%
QOL HF patients + other terminal illnesses	1	192	0.28 [0.00 to 0.57]	n/a
Study setting				
QOL hospital	3	237	0.18 [− 0.07 to 0.44]	0%
QOL community	1	192	0.28 [0.00 to 0.57]	n/a
QOL hospital and community	3	295	0.58 [0.05 to 1.12]	77%
Length of follow-up				
QOL F/u to 12 weeks	7	724	0.38 [0.09 to 0.66]	71%
Patient satisfaction with end-of-life care (PSEOLC)	4	1290	0.39 [0.14 to 0.64]	75%
Patient population				
PSEOLC HF patients + other terminal illnesses	4	1205	0.39 [0.14 to 0.64]	78%
Study setting				
PSEOLC hospital	2	765	0.49 [− 0.03 to 1.01]	92%
PSEOLC community	1	297	0.22 [0.00 to 0.45]	n/a
PSEOLC hospital and community11	1	143	0.37 [0.03 to 0.70]	n/a
Length of follow-up				
PSEOLC F/u to 12 weeks	3	712	0.45 [0.11 to 0.80]	80%
PSEOLC F/u to 24 weeks	1	493	0.23 [0.05 to 0.41]	n/a
Quality of end-of-life communication (QEOLC)	4	995	0.29 [0.17 to 0.42]	0%
Patient population				
QEOLC HF patients	1	80	0.48 [0.03 to 0.92]	n/a
QEOLC HF patients + other terminal illnesses	3	915	0.28 [0.15 to 0.41]	0%
Study setting				
QEOLC hospital	4	995	0.29 [0.17 to 0.42]	0%
Length of follow-up				
QEOLC F/u to 4 weeks	3	483	0.33 [0.09 to 0.57]	20%
QEOLC F/u to 24 weeks	1	512	0.29 [0.12 to 0.47]	n/a

*F/u* follow-up, *HF* heart failure, *n/a* not applicable, *PSEOLC* Patient Satisfaction with End-of-Life Care, *QEOLC* quality of end-of-life communication, *QOL* quality of life

## DISCUSSION

### Principal Findings

This literature review was the first systematic evaluation of the evidence base for the effect of ACP on end-of-life care outcomes in heart failure. The results of the meta-analyses from 14 RCTs including 2924 participants indicated that ACP improved.

- Quality of life (SMD, 0.38; 95% CI [0.09 to 0.66])
- Patient satisfaction with end-of-life care (SMD, 0.39; 95% CI [0.14 to 0.64])
- Quality of end-of-life communication (SMD, 0.29; 95% CI [0.17 to 0.42]) in heart failure compared to usual care

Several sensitivity and subgroup analyses confirmed the direction of the effect. ACP alone compared to ACP as part of a palliative care programme might be equivalent in its effectiveness. ACP that included not only patients but also family members, offered follow-up occasions and considered ethnic preferences was more effective than ACP that did not cover these criteria.

### Comparison with Other Reviews

At the time of writing, this review was the first to have analysed the effect of ACP on outcomes in heart failure in a meta-analysis with a consideration of heterogeneity and characteristics of intervention effectiveness. Several reviews had investigated the effectiveness of interventions to implement ACP but did not investigate the impact of ACP on heart failure.^17–21, 23–26, 36^ Some of these reviews conflated an analysis of the impact of ACP on end-of-life outcomes with the effect of interventions to promote the implementation of ACP.^26^ Data on the actual impact of ACP on end-of-life outcomes was only presented descriptively and missing for heart failure.^26, 36^

### Strengths and Limitations

The strength of this review and meta-analyses consisted of the use of a robust search strategy, assessing the overall quality of the evidence with the GRADE system, rating risks of biases with the Cochrane risk of bias evaluation tool, statistically pooling data for each outcome, performing sensitivity analyses and exploring causes of heterogeneity and intervention effectiveness. Furthermore, we used random effects models and SMDs to account for variations in types of interventions and outcomes measures across studies.

The heterogeneity in studies was mainly due to different study populations combining heart failure patients with patients suffering from other life-limiting illnesses, different study settings, follow-up periods and a lack of standardization of using outcomes measures. The median age of participants was below the median age of people dying from heart failure in the general population. The median follow-up periods for clinical outcomes were comparatively short. It appears unclear whether the positive effect of ACP might mitigate over time. The majority of studies originated from the US but a sensitivity analysis comparing effect sizes of US-based studies with studies from other countries did not show a favourable effect dependent of the health care setting.

### Causes of Clinical Heterogeneity

Heterogeneity is to be expected when aggregating evidence on the effect of complex interventions like ACP.^62^ ACP in itself tends to consist of multiple components and is implemented differently and in different settings.^63^ For example, the timing of offering ACP as an intervention in relation to the patient’s disease trajectory varied in included studies. While most patients in the meta-analysis suffered from heart failure class III or IV of the New York Heart Association, ACP was initiated at different time points: during an admission to hospital,^58^ after a recent hospitalization,^55^ after a deterioration in the patient’s health status^52^ or before a routine clinic appointment with a cardiologist.^53^ These findings on the timing of ACP agree with the published literature which cites a change or deterioration in the patient’s condition, a routine clinical review or a change in a patient’s personal circumstances such as moving into a care home as possible triggers for ACP.^13^

Control patients tended to receive only usual medical practice but no ACP unless it was specifically requested.^52, 54, 56^ The majority of studies provided similar amounts of time for control patients,^42, 49–53, 55, 59, 61^ for example, through social calls consisting of conversational topics unrelated to clinical issues. Therefore, it seems likely that the ACP intervention and not just time made a difference to clinical outcomes. Two studies did not provide a clear description on whether an attempt was made to provide similar amounts of time with patients and the families of the control groups.^57, 58^

### Characteristics of ACP interventions

Our challenge was to constructively explore existing levels of heterogeneity and identify intervention components which may make the use of ACP more effective:

Sidebottom et al.^60^ attributed the success of their ACP intervention to using a trained ACP facilitators, educating patients in communicating their end-of-life care preferences, involving a family member and offering follow-up appointments. The literature affirmed the importance of using trained facilitators and designing multiple ACP components to improve patient outcomes.^63–65^ Similarly, Wong et al.^61^ thought that improvements in their QOL outcomes were due to a comprehensive palliative care programme including multiple ACP components: working as a multidisciplinary team, clear referral guidelines and involvement of carers and patients alike. Their approach is confirmed by the literature that indicates that ACP is more effective as a complex versus a single intervention.^15, 65, 66^

By contrast, Hopp et al.^58^ used the design of a single component ACP intervention without any follow-up, involvement of family members or other healthcare professionals. Additionally, their trial population consisted mainly in African Americans. Studies have shown that this patient population prefers curative rather than palliative care approaches and is less likely to engage with ACP.^67, 68^ A lack of consideration of ethnic preferences, missing follow-up and the exclusion of family members may well have contributed to the low effect of their ACP intervention. The low effect size for ACP in the study by Denvir et al.^53^ was mainly due to the study design, i.e. a feasibility RCT. The data generated in this small cohort was not intended to be sensitive or specific enough to observe a positive effect of the intervention for QOL. Additionally, a high mortality rate during the follow-up period further limited the utility of their data as a trial end point.

The study by Detering et al.^54^ cited five factors for improving patient satisfaction with end-of-life care^1^: using trained facilitators,^2^ conducting a patient-centred discussion,^3^ the involvement of the family,^4^ correctly filing and communicating the ACP documentation with all parties, and^5^ a systematic education of doctors in communicating ACP. Likewise, the study by Engelhardt et al.^56^ delivered ACP through trained care coordinators who had an institutional identity and clinical credibility. Patients could schedule extra meetings with their care coordinator if they had further questions and follow-up was provided. The importance of using respected, senior clinicians to improve the uptake of an intervention like ACP is highlighted in the literature.^69, 70^

Lower effect sizes for patient satisfaction with end-of-life care were found in the study by Brumley et al.^52^ While the intervention slightly improved outcomes, the authors thought that a major cause for the low effect size was the high mortality rate of the study population before follow-up. Their results suggested that providing a multidisciplinary team *early* in the disease trajectory of the patient had a small but positive effect on patient satisfaction with end-of-life care.

Possible causes for the success of the study by Doorenboss et al.^55^ corresponded with factors found in other studies of higher effect sizes: (a) patients were educated by a nurse through a question prompt list before their appointment with the cardiologist, (b) the nurse helped patients identifying and clarifying their care wishes and (c) the clinician was trained in end-of-life communication skills and had an advanced awareness of the patient’s care preferences.

Lower effect sizes for the quality of end-of-life communication were reported in the study by Briggs et al.^51^ Possible causes for the comparatively small effect might have been similar to the factors described in the study by Hopp et al.^58^: ACP was delivered as a single intervention in the context of a 2-h meeting. Interdisciplinary working with other medical specialties was missing, and no follow-up was offered to the patient.

### Potential Reasons for Over- and Underestimation of Effect

To evaluate potential overestimation of the impact of ACP on patient outcomes, we carried out subgroup analyses to compare the effect of ACP as a standard intervention versus ACP as part of a complex palliative care programme. Sufficient data were only available for quality of end-of-life communication, and for this outcome, no difference in effect for ACP as a standard intervention compared to ACP as part of a palliative care programme was observed. Heterogeneity was insignificant and confidence intervals of these two subgroups overlapped substantiating the direction of effect.

The nature of the intervention made it often impossible to blind participants to the intervention resulting in performance and selection biases. These factors may have resulted in a more beneficial effect of ACP compared to standard care since both patients and physicians may believe that the intervention works. These biases are a known issue in assessing complex interventions in palliative care research and need to be considered when interpreting the reliability and statistical significance of effect sizes.

Due to the assumptions to make the conversion from SMDs to ORs, the results are only an approximation. However, the clinical gain seems sufficiently large to justify the effort of engaging with ACP in heart failure.

## CONCLUSIONS AND CLINICAL IMPLICATIONS

Patients suffering from heart failure have significant palliative care needs. Only the minority of those dying from heart failure receives palliative care and their management remains suboptimal. ACP is widely advocated as a way of addressing these care needs. But until now, the effect of ACP on heart failure has not been systematically assessed.

The evidence from this review indicates that ACP improves the quality of life (SMD, 0.38; 95% CI [0.09 to 0.66]), patient satisfaction with end-of-life care (SMD, 0.39; 95% CI [0.14 to 0.64]) and the quality of end-of-life communication (SMD, 0.29; 95% CI [0.17 to 0.42]) for patients suffering from heart failure. Based on findings from 14 RCTs, we suggest the following considerations to facilitate a better engagement with ACP in heart failure:

- Introduce ACP at a significant milestone in the patient’s disease trajectory, for example, after an unscheduled hospital admission, before hospital discharge or after a deterioration in the patient’s health status
- Offer follow-up appointments, preferably two or three meetings or points of contact over a period of time to allow for the clarification and adjustment of care choices
- Be mindful of ACP preferences
- Offer the involvement of family members or of a health care proxy
- Work in a multidisciplinary team and not in isolation within a single medical specialty

## Data Availability

All data generated or analysed during this study are included in this published article and its supplementary information files.
